# Demographics, Clinical Characteristics, and Management Strategies of Epilepsy in Saudi Arabia: A Systematic Review

**DOI:** 10.7759/cureus.63436

**Published:** 2024-06-29

**Authors:** Samer A Almuqairsha, Faisal A Al-Harbi, Alwleed M Alaidah, Turki A Al-Mutairi, Emad K Al-Oadah, Areen E Almatham, Fahad M Alharbi, Albara N Almoshaigah

**Affiliations:** 1 Internal Medicine, Qassim University, Buraydah, SAU; 2 College of Medicine, Qassim University, Buraydah, SAU

**Keywords:** review, saudi, epilepsy, strategy, management, characteristics

## Abstract

Epilepsy accounts for a large part of the global burden of neurological disorders. This review aimed to assess the demographics, clinical characteristics, and management of patients with epilepsy in Saudi Arabia based on studies published from 2018 to 2023.

A systematic review was carried out using PubMed, Medline, Embase, and Cochrane Library from January 2018 to January 2023, where key terms related to the epidemiology, clinical characteristics, treatment, and management strategy of epilepsy in Saudi Arabia were used to search for related studies. All relevant articles published in this period in the English language were included, and data about authors, year of the study, sample size, study design, demographic characteristics, clinical characteristics, and treatment strategy were collected.

A male preponderance, a 6-24.9% family history of epilepsy, an equal distribution of focal and tonic-clonic epilepsy, EEG abnormalities of 19.7-70%, and a higher prevalence of monotherapy regimens were the main findings of this review.

## Introduction and background

Epilepsy is a neurological illness characterized by a persistent predisposition to epileptic seizures and by the associated neurological, cognitive, psychological, and social effects [1]. It represents around 9.9% of the worldwide burden of neurological illnesses and is the most common non-infectious neurological ailment [2].

Around 50 million people worldwide suffer from epilepsy, according to estimates from the World Health Organization [3], with 4.7 million living in the Eastern Mediterranean region at the moment [4]. These high numbers highlight the serious health risks associated with epilepsy and the necessity of receiving the right care. The higher risk of early death among epileptic patients relative to the general population is a concerning feature of epilepsy [5].

All age groups are impacted by epilepsy, which has detrimental effects on the patients, their families, and the healthcare system [6]. Additionally, epilepsy patients experience social, emotional, mental, and physical effects [7]. Even when their epilepsy is under control, individuals with epilepsy nevertheless face higher rates of early death and disability than the general population, and many of them also report a lower quality of life [8]. Furthermore, among all the neurological illnesses, epilepsy has been linked to the greatest rates of disability-adjusted life years in both sexes [9].

About 60% of instances of epilepsy have an unidentified primary cause [10]. Epilepsy can be caused by hereditary or genetic disorders, as well as severe brain injury, stroke, and sequelae from previous illnesses; in many cases, both triggers are involved [11]. The 2010 International League against Epilepsy (ILAE) study classified the etiology of epilepsy as systemic, inherited, and unknown [12].

For epilepsy, antiseizure medication (ASM) (formerly referred to as anti-epileptic drug) therapy is regarded as the accepted standard of care. There are currently over 20 ASMs on the market, the majority of them have been created and put to good use for a long time. The chemical makeup and modes of action of these ASMs vary [13]. When patients with epilepsy take their ASMs as prescribed, their seizures are usually well-controlled and benign, but the psychological and social problems that arise after receiving an epilepsy diagnosis can be far more damaging than the actual seizures [14]. To address the psychological and social problems associated with epilepsy, physical management should be paired with educational intervention [15].

According to estimates, there were 6.54 epileptic patients for every 1000 people in Saudi Arabia in 2015 [16]. Regarding ASM, the findings of an earlier study revealed that drug-resistant epilepsy affected 45% of the Saudi participants [17]. A proportion that was concerningly greater than the frequency of drug-resistant epilepsy documented in other studies [18,19].

This review aimed to assess the demographic, clinical characteristics, and management strategy of epilepsy in Saudi Arabia according to studies published from 2018 to 2023.

## Review

Methods

The reporting of this systematic review was guided by the standards of the Preferred Reporting Items for Systematic Review and Meta-Analysis 2020 (PRISMA 2020)statement.

Search Strategy

The present systematic review was carried out using PubMed, Medline, Embase, and Cochrane Library from January 2018 to January 2023. Key terms related to epidemiology, demographics, clinical characteristics, treatment, and management strategy of epilepsy in Saudi Arabia were used to search for related studies. The abstracts of all related articles addressing the epidemiology, clinical characteristics, treatment, and management strategy of epilepsy were reviewed. The reference lists of included articles and recent reviews, which dealt with the same subject were examined.

Inclusion Criteria

All relevant articles that were published from January 2018 to January 2023 in the English language with the purpose of addressing the clinical characteristics, treatment, and management strategy of epilepsy were included.

Exclusion Criteria

**Figure 1:**
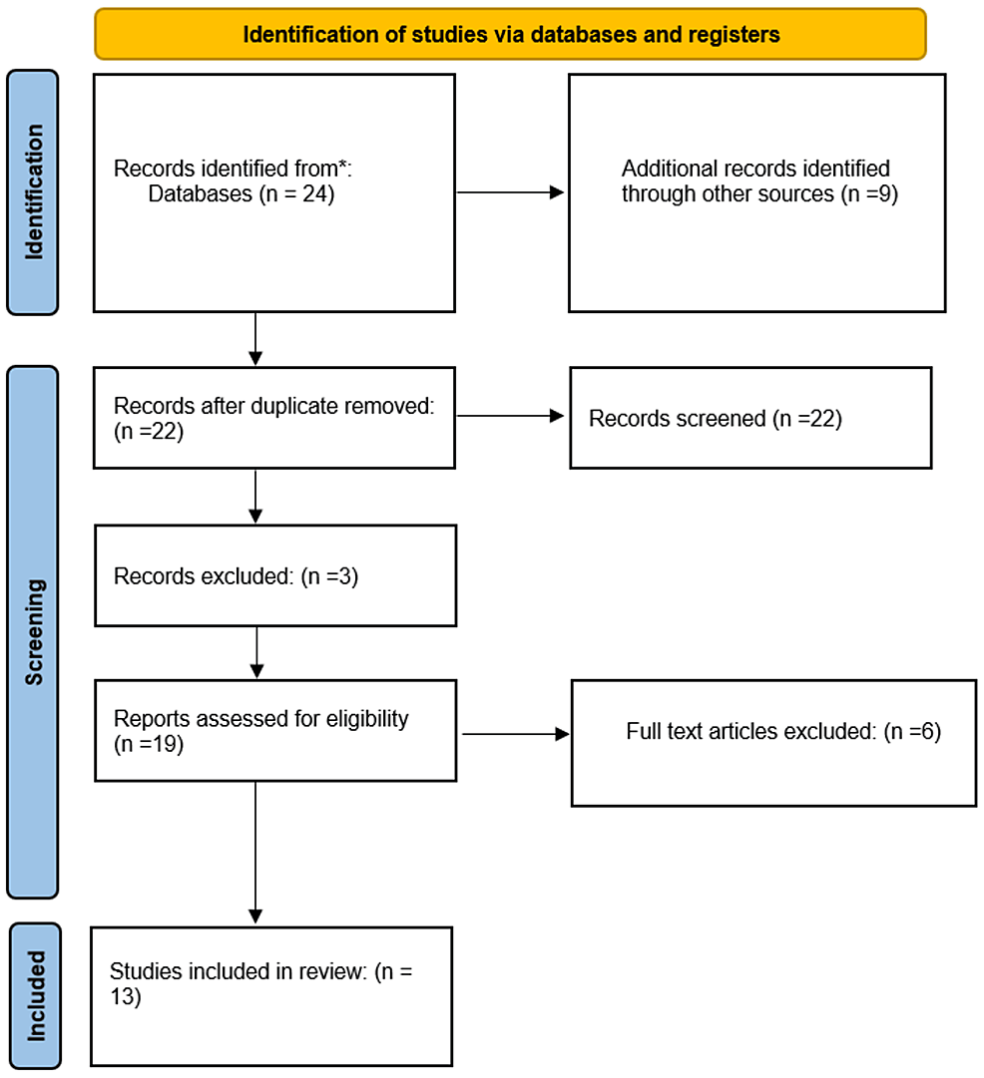
PRISMA flow diagram for the inclusion and exclusion criteria of articles PRISMA: Preferred Reporting Items for Systematic Review and Meta-Analysis

All reviews, duplicate publications, and studies done without an assessment of the clinical characteristics, treatment, or management strategy of epilepsy were excluded. The detailed search strategy and inclusion and exclusion criteria for articles are illustrated in Figure 1.

Data Extraction

One investigator extracted data regarding the authors, year of the study, sample size, study design, demographic characteristics, clinical characteristics, and treatment strategy. Another investigator independently reviewed the accuracy of the extracted data.

Assessment of the Studys' Risk of Bias

Two investigators independently evaluated the methodological quality of the selected studies. Pre-specified questions for each study design were used to measure the risk of bias, and studies with a high risk of bias were not included [20].

Results

Our review included 13 articles covering 3801 people with epilepsy (PWE). Only four studies were retrospective [11,21-23] and nine were cross-sectional [17,24-31].

Demographics

All studies assessed the general characteristics of patients with epilepsy. As for the patient's age, it ranged from 0 to 90 years and 8 studies showed a male predominance [11,17,21,22,25,26,28,31]. According to the family history of epilepsy, it was seen only in five studies [17,22,24,25,30] and showed a prevalence ranging from 6% to 24.9% [22,30].

Only six studies assessed the educational level of the participants; of these, four showed a predominance of high-school educational level [24,26,29,31]. While the predominance was for bachelor’s degrees of education in two studies [27,30].

Table 1 summarizes the demographic characteristics of each study included.

**Table 1:** Reviewed studies according to author information, year of publication, study design, sample size, and demographic characteristics

No.	Authors	Study design	Sample size	Age	Gender	family history of epilepsy	Educational level	Marital status	Employment status
1	Magadmi et al., 2023 [17]	Cross-sectional	101	The mean age was 7.3 years.	67.3% were males.	6.9% had a family history of epilepsy.	Information is not available.	Information is not available.	Information is not available.
2	Alkoblan et al., 2023 [24]	Cross-sectional	246	The mean age was 33.2±12.8 years.	61% were females. 39% were males.	24% had a family history of epilepsy.	54.5% had a high school education or above.	Information is not available.	42.3% were unemployed.
3	Almuqbil et al., 2023 [25]	Cross-sectional	61	52.5% were full-term. The median gestational age was 38 weeks.	57.4% were males.	21.3% had a family history of neonatal seizures.	Information is not available.	Information is not available.	Information is not available.
4	Asiri et al., 2022 [26]	Cross-sectional	200	The mean age was 23 years. 51% of patients were 19-40 years old. 70% were adults.	54.5% of patients were males.	Information is not available.	27% had graduated from high school, and 26% had a bachelor's degree.	46% were single. 20% were married.	Information is not available.
5	A. Alharbi et al., 2022 [27]	Cross-sectional	70	64% had 20-30 years.	51% were females.	Information is not available.	50% had a bachelor’s degree, 26% had a high school/diploma, and 23% had an elementary/intermediate school.	Information is not available.	Information is not available.
6	Alhaidari et al., 2022 [28]	Cross-sectional	524	58% were 21 to ≤40 years old.	57.3% were males.	67% had structural epilepsy. 15.1% had a genetic cause.	Information is not available.	Information is not available.	Information is not available.
7	Almohammed et al., 2021 [29]	Cross-sectional	126	36.5% had 21-30 years. 88.9% were younger than 50 years.	67.5% were females.	Information is not available.	42.1% had a high school education.	Information is not available.	Information is not available.
8	Alshurem et al., 2021 [21]	Retrospective	1151	The mean age was 31 years.	53% males and 47% females.	Information is not available	Information is not available	Information not available.	Information is not available.
9	Alshamrani et al., 2020 [30]	Cross-sectional	546	42.9% were 22-30 years.	52.9% were females.	24.9% had a family history of epilepsy.	56.2% had a Bachelors education.	Information is not available.	Information is not available.
10	Alsfouk et al., 2020 [22]	Retrospective	201	The median age at treatment initiation was 73 years.	57.2% were males.	6% had a family history of epilepsy.	Information is not available.	Information is not available.	Information is not available.
11	Shahid et al., 2018 [23]	Retrospective	184	Age was 12 -85 years (mean 35.4±19.5 years).	50.5% were females.	Information is not available.	Information is not available.	Information is not available.	Information is not available.
12	Alonazi et al., 2018 [11]	Retrospective	221	52.9% had an age at presentation of 1–24 months.	60.2% were males.	Information is not available.	Information is not available.	Information is not available.	Information is not available.
13	Al Taho et al., 2018 [31]	Cross-sectional	170	The mean age was 38.7 years (range 18-90 years).	100% were males.	Information is not available.	77.1% had high school and above education. 24.7% were students.	24.7% were unemployed.	Information is not available.

Clinical Characteristics and Management Strategy

As for the clinical characteristics of patients with epilepsy included in this systemic review, five studies showed a higher prevalence of generalized tonic-clonic epilepsy type [11,17,23,26,31], five studies showed a predominance of focal epilepsy type [21,22,25,27,28], and three studies did not assess the epilepsy type [24,29,30].

Only five studies assessed the frequency of the seizures, where 68.7% of patients in the Alkoblan et al. 2023 study had seizures on a yearly basis [24], 33% had monthly seizures in the Asiri et al. 2022 study [26], and 61.4% had up to three seizures per year in the A. Alharbi et al. 2022 study [27]. At the same time, 48.1% were seizure-free over the previous 3 months in the Alshamrani et al. 2020 study [30] and 57.7% had ≤5 seizures before treatment [22].

Of the 13 studies included in this review, only 5 studies assessed the EEG findings of the patients with epilepsy included. Almuqbil et al. (2023) showed that 47.5% of patients had EEG abnormalities [25], Alhaidari et al. (2022) found that 70% had EEG abnormalities [28], and Alshurem et al. (2021) showed that 43% had abnormal EEG findings [21]. Alsfouk et al. (2020) reported the lowest prevalence of EEG abnormalities, which was 19.7% [22].

Nine studies assessed the management strategy of the studied patients. Of these studies, five studies showed a higher prevalence of monotherapy regimens [17,21,22,25,26], three studies showed a higher prevalence of polytherapy anti-epileptic medicine (AEM) regimens, while three studies did not assess the management strategy [27,28,31].

Shahid et al. (2018) [23] showed that 61% of patients with epilepsy had idiopathic/cryptogenic epilepsy, and Al Taho et al. (2018) [31] found that 90.6% had an idiopathic cause and 5.3% had cryptogenic epilepsy.

The study done by Alhaidari et al. (2022) [28] showed that 67% had structural epilepsy, and the study done by Alonazi et al. (2018) [11] showed that 43% of patients had structural/metabolic epilepsy and 44% had unknown epilepsy. MRI neuroimaging was done only in two studies [21,28], where 30% of patients with epilepsy had abnormal MRI findings in the first study as compared to 70% in the second study.

Table 2 summarizes the clinical characteristics and antiseizure medication regimen of each study included.

**Table 2:** Reviewed studies according to the patient's clinical characteristics and antiseizure medication regimen

No.	Authors	Seizures frequency and duration	Epilepsy type and characteristics	Medication duration and complications	Characteristics of antiseizure medications	EEG abnormalities and MRI findings
1	Magadmi et al., 2023 [17]	Information is not available.	45.5% had generalized tonic-clonic epilepsy.	39.6% were on medication for more than two years. Electrolyte disturbance was reported in two-thirds of the patients, liver function test disturbance in 40%, and cognitive or motor delay in <20%.	61.4% were on monotherapy ASM. The most common drugs were levetiracetam and valproic acid.	Information is not available.
2	Alkoblan et al., 2023 [24]	3.3% suffered from seizures on a daily basis, and 68.7% on a yearly basis.	Information is not available.	Information is not available.	96.3% were on anti-epileptic treatment, mostly levetiracetam (52.5%) and valproic acid (26.7%).	Information is not available.
3	Almuqbil et al., 2023 [25]	43% had epilepsy episodes.	The most common etiologies were hypoxic-ischemic encephalopathy (42.6%) and intracranial hemorrhage (19.7%). 47.5% had EEG abnormalities. 36.1% had focal epilepsy. 23% had clonic seizures.	Information is not available.	57% were on monotherapy.	Information is not available.
4	Asiri et al., 2022 [26]	The duration of epilepsy was 1-5 years or >10 years in 34% of patients. 33% had monthly seizures, and 26% were free during the last year.	33% had symptomatic epilepsy, 36.5% had genetic generalized epilepsy, and 30.5% had seizures of unknown etiology. 53% had generalized seizures; of these, 44% had generalized tonic-clonic or focal to bilateral tonic-clonic seizures, and 46% had focal seizures.	Information is not available.	39.5% were on monotherapy. 60.5% were on two or more medications.	Information is not available.
5	A. Alharbi et al., 2022 [27]	61.4% had up to three seizures per year.	55.7% had focal seizures. 44.3% had generalized seizures.	Information is not available.	51.4% were on two or more medications and 48.6% were on one medication. 90% were adhering to and regularly using their medication.	Information is not available.
6	Alhaidari et al., 2022 [28]	Information is not available.	59.8% had focal epilepsy, 12.1% had generalized epilepsy.	Information is not available.	54.5% were on polytherapy. 41.5% were on monotherapy. 4% were on no treatment.	70% had EEG abnormalities and 62% had abnormal MRI findings.
7	Almohammed et al., 2021 [29]	Information is not available.	Information is not available.	Information is not available.	Information is not available.	Information is not available.
8	Alshurem et al., 2021 [21]	Information is not available.	66% had focal epilepsy and 34% had generalized epilepsy.	Information is not available.	68.8% were on monotherapy. 31.2% were on polytherapy.	MRI revealed lesions in 30% and 43% had abnormal EEG findings and showed generalized epileptiform discharges in 10%. Extratemporal epileptiform discharges were present in 16% and temporal epileptiform discharges in 16%.
9	Alshamrani et al., 2020 [30]	48.1% had been seizure-free over the previous 3 months.	Information is not available.	77.2% complained about their treatment plan.	Information is not available.	Information is not available.
10	Alsfouk et al., 2020 [22]	57.7% had ≤5 seizures before treatment, 26.4% had more than 20 seizures. 78.6% remained seizure-free at the last follow-up.	99.5% had focal epilepsy, 38.8% had focal seizures, 35.3% had focal to bilateral generalized tonic-clonic seizures, and 25.4% had both focal and focal to bilateral tonic-clonic seizures.	The median ASM therapy follow-up duration was 7.5 years.	91.5% were on monotherapy, and 8.5% were on a combination of two ASMs. 37.8% were on lamotrigine, 24.8% were on valproate, 14.4% were on carbamazepine, and 6.4% were on levetiracetam. 66.7% were taking concomitant drugs.	19.7% had EEG epileptiform abnormalities.
11	Shahid et al., 2018 [23]	Information is not available.	61% had idiopathic/cryptogenic epilepsy, 83% had generalized seizures, and 14% had focal seizures.	Information is not available.	Information is not available.	70% had abnormal EEGs and 41% had focal to bilateral EEG abnormalities.
12	Alonazi et al., 2018 [11]	Information is not available.	47.9% had generalized tonic-clonic seizures, 6.7% had focal seizures, 57.6% had generalized tonic-clonic seizures, 90.6% had idiopathic causes, and 5.3% had cryptogenic epilepsy.	Information is not available.	Information is not available.	61.5% had abnormal EEG findings.
13	Al Taho et al., 2018 [31]	Information is not available.	Information is not available.	Information is not available.	61.2% were on polytherapy. 37.6% were on monotherapy and 1.2% were on no treatment.	Information is not available.

Discussion

Thirteen publications that evaluated the clinical traits and approach to treating epilepsy in Saudi Arabia were included in this review. Patients with epilepsy in these trials ranged in age from 0 to 90 years. Ages ranged from 41 to 44 years old, according to other studies [32-34].

According to this review, men are more likely than women to have epilepsy [11,17,21,22,25,26,28,31]. Previous investigations [32,35-37] revealed a similar male predominance. This discrepancy was ascribed to the fact that men are more likely than women to have head injuries and that women tend to hide their epilepsy diagnosis [35-37]. This result, however, was at odds with earlier research on the Arab community that indicated an incidence of epilepsy that favored women. This distinction was also made clear by epidemiological research conducted on the Arab community, which suggested that the gender discrepancy may have its roots in community differences [38,39]. Other worldwide research [40,41] also revealed the same female majority. There are hints that women may be more inclined to hide their illness since it would have resulted in social stigma and made marriage more difficult [40,41].

In the included studies, the percentage of people with a family history of epilepsy varied from 6% to 24.9% [22,30]. This prevalence range is in line with national studies, such as the one carried out in Al Qassim in 2001 [42] as well as global research [43,44].

This review concluded that there was a similar predominance of focal epilepsy and generalized tonic-clonic epilepsy types. Five studies found that the prevalence of focal seizures was higher than that of generalized tonic-clonic seizures. It was also demonstrated by Khreisat et al. that the most prevalent kind of seizure in structural/metabolic epilepsy was generalized tonic-clonic seizures. Caregivers and doctors may occasionally fail to notice the onset of seizures, which may have begun as focal seizures before becoming generalized, which explains the high prevalence of generalized epilepsy [45]. Banerjee et al. highlighted the higher prevalence of generalized onset seizures in underdeveloped nations by reporting focal onset seizures ranging from 20% to 66% in a review [41]. These authors suggest that uncertainties in the application of the classification and a lesser level of diagnostic expertise may account for these variations [41].

Moreover, Garcia-Martin et al. found that focally onset epileptic seizures accounted for the majority of seizures (75.5%) as opposed to generalized seizures (17.5%) [46]. In a study of adults with epilepsy (mean age, 31.5 years), Rezaeian et al. found that widespread crises (78%) were more common than focal crises (22%) [47]. Similar to our data, tonic-clonic seizures were common among the generalized crises. This result was in line with prior research involving Asian and African studies [48,49]. However, other studies observed a higher proportion of focal seizures [32,50,51]. This discrepancy was ascribed to patients' and observers' inadequate detection of the early focal symptoms, which causes them to miss the onset and become aware of the patient's broad convulsion [52]. Meanwhile, some of the reported generalized seizures may have been focal seizures that progressed to bilateral tonic-clonic seizures. Cultural concerns may have contributed to an underreporting of focal seizures [53].

According to Shahid et al. (2018) [23] and Al Taho et al. (2018) [31], 90.6% of patients with epilepsy had idiopathic causes of epilepsy and 5.3% had cryptogenic epilepsy. These findings are presented in this review. This predominance was also noted in earlier research, which reported a high frequency of idiopathic epilepsy [3,54].

Sixty-seven percent (67%) of patients in this review had structural epilepsy, according to a study by Alhaidari et al. (2022) [28]. Another study by Alonazi et al. (2018) [11] revealed that 43% of patients had structural/metabolic epilepsy and 44% had unexplained epilepsy. Kroczka et al. found similar findings, demonstrating the prevalence of structural/metabolic epilepsy, formerly termed symptomatic epilepsy [55]. Typically, organic brain lesions presenting with neurological abnormalities, developmental delay, and abnormal EEG cause this kind of epilepsy [56]. The most frequent cause of structural/metabolic epilepsy, according to research by Kroczka et al. [55], was perinatal insults, primarily hypoxia ischemic encephalopathy. Metabolic disorders, infections, and trauma were the next most common causes. Corresponding to this, earlier research [56] examined a variety of causes with varying frequency such as intracranial infection, cerebral deformity, degenerative brain illness, and brain damage sustained during neonatal life.

Based on the AEM utilized in this research, three studies demonstrated a greater incidence of polytherapy AEM regimen and five studies demonstrated a higher prevalence of monotherapy AEM regimen [17,21,22,25,26]. Three studies did not evaluate the management strategy that was employed [27,28,31]. A prior study found that monotherapy AEM regimens were more common and that the most often prescribed medications were carbamazepine, valproic acid, phenobarbital, and lamotrigine [32]. This agrees with the present review where the most commonly reported drugs in studies involved in this review were levetiracetam, valproic acid, lamotrigine, valproate, and carbamazepine.

Studies have shown that 20% to 30% of patients with epilepsy had trouble controlling their epilepsy [57]. Furthermore, in other investigations, the degree of control over epileptic seizures was not altered by the administration of novel ASMs [58,59]. In earlier research, monotherapy was employed at a frequency of 48.6% to 60% [47,50,51].

According to the current analysis, there have only been two studies that used MRI neuroimaging [21,28], with the first study showing 30% of patients with epilepsy with aberrant MRI findings and the second study showing 70%. It was discovered that neuroimaging might investigate the connection between anomalies in brain activity and the anatomical locations of brain pathologies [60]. Determining the cause of symptomatic epilepsy and the degree of related disease is also helpful. Nonetheless, certain anomalies, such as focal cortical dysplasia, became apparent with age and were not initially identified by neuroimaging. In a similar vein, a recent study conducted in Saudi Arabia revealed that children who experienced their first apparent seizure had a significant frequency of abnormalities (42.7%) on brain CT scans [61]. This implies that when a youngster presents with their first seizure, emergency CT should be taken into consideration. The Arteaga-Rodríguez et al. investigation reported the utilization of at least one brain imaging test, with positive results between computed tomography and magnetic resonance imaging of the skull of 48.6% and 77.9%, respectively [32]. It has been established that magnetic resonance imaging is the best imaging modality for treating epilepsy [62].

For most epileptic patients, electroencephalography (EEG) is a practical and economical investigative method [63]. The classification of seizure types, the identification of a particular condition, and the subsequent long-term prognosis prediction can all be aided by EEG [60]. The EEG results of the patients with epilepsy were evaluated in just five trials included in this analysis, with EEG abnormalities ranging from 19.7% to 70%. Furthermore, this review's spectrum of EEG abnormalities is consistent with that of earlier research [32,47].

Summary

In the 13 studies that were reviewed, there was a male preponderance in 9 studies, and between 6% and 24.9% of patients had a family history of epilepsy. Five studies suggested focal epilepsy was more common, and five suggested generalized tonic-clonic epilepsy was more common. EEGs showed abnormalities in the range of 19.7% to 70%. Five studies showed a higher prevalence of monotherapy regimens, and three showed a higher incidence of polytherapy. Only two investigations used MRIs, and the results revealed 30% and 70% of anomalies. The studies had various aims, and patient demographics and clinical and management data were included in most of the studies. However, several studies lack certain data, as this is related to some clinical and treatment regimens of epilepsy because gathering this information was not the main objective of the study.

## Conclusions

This review discussed the clinical and demographic characteristics of patients with epilepsy in 13 studies published from 2018 to 2023, which included 3801 epileptic patients. Future studies that assess all the demographic and clinical data about patients with epilepsy in Saudi Arabia are needed for a better understanding of the magnitude of this health issue.
